# Long-term tolerance and efficacy of venom immunotherapy after an episode of Crohn’s disease and ankylosing spondylitis after up-dosing 

**DOI:** 10.5414/ALX02526E

**Published:** 2024-10-24

**Authors:** Marius Winkler, Franziska Ruëff, Silvan Lange, Annett Walker, Eva Oppel

**Affiliations:** Department of Dermatology and Allergy, LMU Munich, University Hospital, Munich, Germany

**Keywords:** vespula venom allergy (VVA), anaphylaxis, venom immunotherapy (VIT), allergen-specific immunotherapy (AIT), autoimmune disorders, ankylosing spondylitis, Crohn’s disease

## Abstract

Hymenoptera stings can cause severe anaphylactic reactions in patients with an underlying Hymenoptera venom allergy (HVA). In such cases, venom immunotherapy (VIT) is a highly effective measure to prevent future anaphylaxis. The management of patients with a clear allergological indication for VIT and contraindications to VIT (e.g., autoimmune diseases) remains a clinical challenge. We report the case of a 54-year-old male gardener who experienced life-threatening anaphylaxis after being stung by wasps in the head and neck region. After confirmation of a Vespula venom allergy (VVA) by intradermal test and VV-specific serum IgE antibodies, VIT was started using a rush protocol. One month after reaching the maintenance dose, the patient experienced a worsening of his pre-existing Crohn’s disease and ankylosing spondylitis. VIT was stopped, and the autoimmune diseases were treated with systemic steroids and sulfasalazine. As the patient wished to remain in his profession, and in view of the previous severe anaphylaxis, we restarted VIT after the autoimmune diseases had resolved, using a slower up-dosing protocol. This approach was tolerated without side effects, and the patient tolerated a sting challenge and several field stings without anaphylactic symptoms.

## Introduction 

Hymenoptera venom allergy (HVA) is the most common cause of anaphylaxis in adults [1]. The most common triggers are stings from honeybees (*Apis mellifera*) and wasps of the Vespidae family, less commonly from hornets (*Vespa crabro*) and bumblebees (*Bombus spp*.). Stings can cause systemic symptoms that are life-threatening in some patients [4]. In Europe, the frequency of self-reported anaphylactic sting reactions in adults ranges from 0.3 to 7.5% [2, 3]. 

Venom immunotherapy (VIT) is a highly effective treatment for HVA and prevents future systemic reactions due to Hymenoptera stings [5, 6, 7]. Vespula venom (VV) VIT shows a 91 – 96% protection rate against future systemic Vespula sting reactions [8, 9]. VIT includes an initial build-up phase and a subsequent maintenance phase [10]. By increasing the amount of venom administered subcutaneously, a switch from a pro-allergic to a tolerogenic state of the immune system can be observed [11]. Although the molecular mechanisms underlying VIT are not fully understood, a key role is played by IL-10-producing regulatory T cells (Treg) and regulatory B cells (Breg). Both are upregulated by VIT and suppress the activity of pro-allergic Th2 cells, causing a shift from Th2 to Th1 immune responses, thereby preventing cytokine release, degranulation of mast cells and basophils, recruitment of eosinophils, and induction of venom-specific IgG4 [12, 13, 14]. 

Autoimmune diseases such as chronic inflammatory diseases or autoimmune disorders are considered contraindications to immunotherapy [15]. Current guidelines of the European Academy of Allergy and Clinical Immunology (EAACI) suggest that VIT should not be performed in patients with active multiorgan autoimmune diseases (absolute contraindication), while autoimmune diseases in remission are considered relative contraindications. Furthermore, stable organ-specific autoimmune diseases such as rheumatoid arthritis or Hashimoto’s thyroiditis are not considered contraindications [10]. However, in patients at high risk of exposure and with a history of severe anaphylactic sting reactions, VIT remains the only option to prevent severe anaphylaxis to Hymenoptera sting anaphylaxis [16]. Therefore, even in the presence of contraindications, it may still be necessary to administer VIT to an individual patient. 

## Case report 

We report on a 54-year-old landscape gardener who during his work touched a vespiary while cutting ivy on a wall. He was stung several times in the head and neck. One minute later, he began to develop immediate-type symptoms which eventually led to cardiac arrest. The patient was successfully resuscitated and survived without sequelae. His medical history included Crohn‘s disease and ankylosing spondylitis without symptoms or need for treatment at the time. He was otherwise healthy. There was no history of previous anaphylaxis. 

Skin prick tests with bee and wasp venom (ALK Prick SQ, Hamburg, Germany) at concentrations up to 100 μg/mL were negative. An intradermal test (ALK-lyophilized SQ) with 1 μg/mL VV showed an immediate-type reaction. Serum specific IgE levels were significantly elevated with respect to VV (CAP class 3; 4.42 kU/L) and bee (CAP class 2; 1.27 kU/L) (CAP FEIA, ThermoFisher, Dreieich, Germany). The baseline serum tryptase concentration was within the normal range (2.21 μg/L; 95th percentile < 11.4 μg/L). Skin examination revealed no cutaneous manifestations of mastocytosis. 

Together with the patient‘s medical history, the intradermal test and measurement of VV-specific IgE antibodies confirmed a VV allergy. 

Because of his joint disease, the patient wanted to stay in his job in order to mobilize his joints during physical work. He did not want to give up his outdoor activities, or at least avoid work that was more likely to expose him to stings. VIT with VV (ALK-lyophilized SQ) was started with a 4-day rush protocol. The maintenance dose of 100 μg was easily achieved. 

One month later, the patient developed acute arthritis in several joints, including the subtalar joints, where symptoms were particularly severe. The patient developed abdominal pain and progressive hemorrhagic diarrhea. Colonoscopy revealed inflammatory Crohn’s disease. Laboratory tests showed an elevated CRP concentration (9.77 mg/dL; normal range < 0.9 mg/dL) and an accelerated erythrocyte sedimentation rate (76 mm). The patient was treated with methylprednisolone (initially 100 mg/d) and sulfasalazine (3 g/d), while VIT was discontinued. After remission of clinical symptoms, steroid therapy was gradually tapered. The patient was still unwilling to give up work. VIT was restarted with a conventional build-up protocol using a depot preparation (ALK-Abelló depot SQ), reaching the maintenance dose of 100 µg within 5 weeks. A yellow jacket sting challenge was performed 6 months later and showed no systemic symptoms. Further maintenance treatment and several wasp stings during his work were well tolerated. 

## Discussion 

An association between allergen-specific immunotherapy (AIT) and exacerbation of chronic inflammatory or autoimmune diseases may be possible. In our patient, the acute exacerbation of Crohn’s disease may have been induced by the rapid build-up phase of VIT. However, after symptomatic treatment and later switching to a slower build-up protocol with a depot preparation, the patient tolerated VIT very well. Also in terms of major local and systemic allergic reactions, slower build-up protocols are associated with a lower rate of side effects [24]. As autoimmune diseases are considered a contraindication to VIT, there are no clinical studies on the effect of VIT on autoimmune diseases or vice versa [17]. Therefore, the current knowledge on the possible interaction between VIT and autoimmune diseases is based on a few case reports. The outcome of the underlying disease was highly variable. A small number of case reports described the development of several autoimmune diseases associated with AIT, such as multiple sclerosis, vasculitis or Sjögren’s syndrome. Prior to AIT, affected patients had no previous signs or symptoms of autoimmune disease [18, 19, 20]. Other authors reported rheumatoid arthritis newly diagnosed during bee VIT in a patient who had a low anti-citrullinated protein antibody titer and no clinical symptoms before starting VIT [21]. 

Fujioka et al. [22] reported on 13 patients with allergic rhinitis and underlying rheumatic diseases such as rheumatoid arthritis, Sjögren’s syndrome, eosinophilic granulomatosis with polyangiitis and polymyositis. All patients tolerated AIT well, but 3 patients experienced a temporary worsening of their autoimmune disease. However, a causal relationship with AIT was excluded by the treating physician [22]. Jeßberger et al. [23] reported on 2 patients with ankylosing spondylitis, 1 of whom also had colitis ulcerosa and 1 of whom had lupus erythematosus. Both patients tolerated VIT well and none of them had a worsening of the underlying autoimmune disease [23]. 

However, VIT would only be recommended in selected patients with contraindications such as the one above, in whom the occupational risk of stinging was significantly increased and the patient had a history of a near-fatal sting reaction. In addition, the patient refused to change his occupation because regular outdoor physical activity had a beneficial effect on his ankylosing spondylitis-related joint pain. 

VIT can be performed in selected patients despite contraindications. If VIT is performed in a patient despite contraindications, close monitoring of symptoms related to autoimmune comorbidities or other underlying diseases is recommended. Based on our observations, we would use a slower protocol with a depot-preparation for the up-dosing phase in patients with a history of autoimmune disease. 

## Authors’ contributions 

Hereby, we confirm that all authors made substantial contributions to the conception or design of the work; the acquisition, analysis, and interpretation of data; and drafting the work and revising it critically. All authors approved the final version. 

## Funding 

The authors received no funding for this case report. 

## Conflict of interest 

FR was a member in advisory boards for ALK-Abelló Arzneimittel GmbH, Blueprint medicines (Germany), and received payments for lectures from ALK-Abelló Arzneimittel GmbH Novartis, and ThermoFisher. MW received payments for lectures from ALK-Abelló Arzneimittel GmbH. The other authors declare that there is no conflict of interest. 
